# A Growing Concern for Cashew and an Unexpected Risk From Almonds: Data From the Anaphylaxis Registry

**DOI:** 10.1111/all.16619

**Published:** 2025-06-13

**Authors:** Veronika Höfer, Sabine Dölle‐Bierke, Dominique Sabouraud‐Leclerc, Amandine Divaret‐Chauveau, Alice Köhli, Maria Breiding, Karin Hartmann, Lars Lange, Nikolaos Papadopoulos, Hagen Ott, Stephanie Hompes, Maria Beatrice Bilò, Blanca E. García, Margitta Worm

**Affiliations:** ^1^ Division of Allergy and Immunology, Department of Dermatology, Venereology and Allergology Charité – Universitätsmedizin Berlin, Corporate Member of Freie Universität Berlin, Humboldt‐Universität Zu Berlin, and Berlin Institute of Health Berlin Germany; ^2^ Presidency on Behalf of Allergy Vigilance Network Vandoeuvre les Nancy France; ^3^ Pediatric Unit University Hospital Reims Reims France; ^4^ UR 3450 DevAH – Department of Physiology, Health Faculty University of Lorraine Vandœuvre‐les‐Nancy France; ^5^ Pediatric Allergy Department Children's Hospital, University Hospital of Nancy Vandœuvre‐les‐Nancy France; ^6^ Division of Paediatric Allergology, Department of Pediatrics Children's Hospital Lucerne Lucerne Switzerland; ^7^ Department of Pediatrics Hospital Baden Baden Switzerland; ^8^ Division of Allergy University Children's Hospital and Children's Research Center, University of Zurich (UZH) Zurich Switzerland; ^9^ Divison of Allergy, Department of Dermatology University Hospital Basel and University of Basel Basel Switzerland; ^10^ Department of Clinical Research University Hospital Basel and University of Basel Basel Switzerland; ^11^ Department of Biomedicine University Hospital Basel and University of Basel Basel Switzerland; ^12^ Department of Paediatrics St. Marien‐Hospital Bonn Germany; ^13^ Department of Allergy, 2nd Paediatric Clinic University of Athens Athens Greece; ^14^ Division of Pediatric Dermatology and Allergology Children's Hospital Auf der Bult Hannover Germany; ^15^ Department of Paediatrics Altona Children's Hospital Hamburg Germany; ^16^ Department of Clinical and Molecular Sciences Università, Politecnica Delle Marche Ancona Italy; ^17^ Department of Internal Medicine/Allergy Unit University Hospital Ospedali Riuniti Ancona Italy; ^18^ Allergology Service Hospital Universitario de Navarra Pamplona Spain

**Keywords:** almond allergy, cashew allergy, European Anaphylaxis Registry, hazelnut allergy, tree nut anaphylaxis

## Abstract

**Background:**

Food allergies are a major health concern with rising prevalence. Dietary habits are changing, and information about cashew‐induced anaphylaxis is limited.

**Methods:**

Cases of tree nut‐induced anaphylaxis (TIA) registered from 2007 until April 2024 were extracted from the European Anaphylaxis Registry and analyzed.

**Results:**

1389 cases of TIA out of 5945 registered food‐induced reactions (23%) were identified. 1,083 cases with confirmed elicitor status, including 845 children (median age 4 years, 61% male) and 238 adults (38 years, 40% male), were selected for further analysis. The most frequent elicitors among children were cashew (*n* = 334), hazelnut (*n* = 211) and walnut (*n* = 146). The proportion of cashew‐induced anaphylaxis increased from 2007 to 2024, and reactions were frequently caused by small amounts (< 1 teaspoon). Adults reacted frequently to hazelnut (*n* = 105), walnut (*n* = 47) but also almond (*n* = 35) and to higher amounts. Potential cofactors were present in 50% of the adult patients and 17% of children. The reaction severity was age‐independent, and only a minority of patients was previously aware of their allergy (children 23%, adults 21%). The use of adrenaline was low in lay treatment (children 13%, adults 3%) and reached approximately 40% upon professional treatment.

**Conclusion:**

Cashew is an increasing, relevant allergen leading to anaphylaxis and is now the most frequent cause of TIA among children. These findings highlight the need for effective prevention and treatment measures. Almond was a frequent elicitor among adults and should be further monitored. The acute management requires improvement to comply with current guidelines.

## Graphical abstract

- Tree nut‐induced anaphylaxis constitutes 23% of food‐induced anaphylaxis among the anaphylaxis registry.
- Confirmed elicitors differed between children (*n* = 845) and adults (*n* = 238): cashew, hazelnut, and walnut were most frequent among children, while hazelnut, walnut, and almond were most frequent among adults.
- Cashew‐induced anaphylaxis increased from 2007 to 2024 among children but rarely affects adults (*n* = 11).

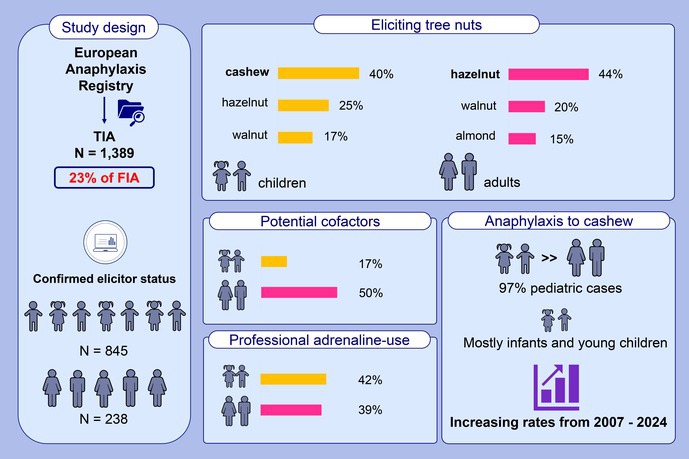

**Abbreviations**
AAI: Adrenaline‐autoinjector
EAR: European Anaphylaxis Registry
ED: Eliciting dose
FIA
: Food‐induced anaphylaxis
LTP: Lipid‐transfer‐protein
NIAID/FAAN: National Institute of Allergy and Infectious Diseases/Food Allergy and Anaphylaxis Network
OIT: Oral immunotherapy
TIA: Tree nut‐induced anaphylaxis

## 1 Introduction

Food allergies affect between 0.8% (challenge proven) and 13.1% (self‐reported) of the European population with an increasing prevalence [1]. Tree nuts are frequent elicitors of food allergy and reach a lifetime prevalence of 0.9% in Europe [2]. Among tree nuts, hazelnut, almond, walnut, pecan nut, cashew, pistachio, Brazil nut, and macadamia nut are most commonly consumed [3]. Even very small amounts can cause severe anaphylaxis in allergic individuals [4]. Thus, tree nuts are among the 14 allergens which have to be declared on food labels, but also on menus in e.g., restaurants and bakeries as defined in the European Regulation 1169/2011, Annex II [5].

Different proteins including 2S‐albumins, vicilins, legumins, Bet v 1‐like proteins (PR‐10 proteins), profilins, oleosins, and lipid transfer proteins (LTP) are the allergenic sources of tree nuts [6]. Tree nut‐induced anaphylaxis (TIA) can occur throughout life [7], but the sensitization patterns are age‐dependent. Sensitizations to storage proteins are common in children while LTP and PR‐10 protein sensitization become more frequent with increasing age [8, 9, 10, 11, 12]. PR‐10 protein‐mediated tree nut allergies do not often cause anaphylaxis, but it can rarely occur in the presence of cofactors [13]. The onset of tolerance in tree nut allergy is possible, but not common [14].

The management of TIA comprises the strict avoidance of tree nuts in the daily diet and the prescription of an emergency medication, including adrenaline‐autoinjectors (AAI) [15]. Oral immunotherapy with tree nuts is a causal treatment option [16], but requires that patients are willing to consume small amounts of their allergen on a regular basis in order to increase or maintain tolerance. Treatment‐related adverse reactions are frequent and mostly local, but systemic allergic reactions including anaphylaxis may occur [16].

We aimed to identify phenotype‐specific features characterized by age distributions, symptoms, and severity of the reaction, the presence of potential cofactors and comorbidities, and to analyze time trends and regional patterns of TIA by analyzing data from the Anaphylaxis Registry. Our data shall support a better clinical understanding of TIA‐affected patients and support measures for improved management.

## 2 Methods

### 2.1 European Anaphylaxis Registry

The European Anaphylaxis Registry (EAR) [17] acquires data from anaphylactic reactions of patients who present in specialized allergy centres across Europe and Brazil within twelve months after a real‐life anaphylactic reaction (reactions during oral food challenges or other deliberate allergen contacts are not included). 142 centres located in fourteen countries participated in the EAR (Germany, Switzerland, Austria, Spain, Italy, Poland, Bulgaria, Greece, Brazil, Romania, Ireland and French‐speaking countries (France, Luxembourg, Belgium) represented by the Allergy Vigilance Network). The study was approved by the ethics committee at Charité—Universitätsmedizin Berlin, Germany (EA1/079/06), and was accredited by the local ethics committees in participating centres. The study is registered on ClinicalTrials.gov (Identifier: NCT05210543). Written informed consent of participants or their caregivers was obtained. Data are allergist‐reported based on the medical history obtained during regular appointments, discharge papers, or other paperwork capturing information on the anaphylactic reaction, entered by trained staff, and checked for plausibility in a data cleaning process including a query process with the reporting centres by the coordinators of the EAR. Only cases that fulfill the criteria of anaphylaxis according to the National Institute of Allergy and Infectious Diseases/Food Allergy and Anaphylaxis Network (NIAID/FAAN) [18] are included in the analysis. Data include information like sex, age, symptoms and circumstances of the reaction, elicitor and cofactors, treatment (lay and physician) and long‐term management. The elicitor status is either unknown, confirmed, or highly suspected. This information is provided by the physician recording the reaction and relies on the outcome of routinely performed diagnostic procedures (e.g., medical history, skin prick test, specific IgE‐antibodies, component‐resolved diagnostics, and/or oral food challenges).

### 2.2 Data Extraction

We filtered the EAR (data reported from 2007 (beginning of data collection) until April 4th, 2024) for the elicitor group “tree nut”, which include hazelnut, cashew, walnut, almond, Brazil nut, macadamia nut, pecan nut, pistachio and the category “other rare/unidentified nuts”, which includes for example reactions to nut mixtures where no single nut was identified as the elicitor. Pine nut seeds were not included in the analysis. Except for the overall cohort description, we focused only on cases with confirmed elicitor status.

### 2.3 Statistical Analysis

Statistical analysis was conducted using R, version 4.3.1 (2023‐06‐16) [19]. Descriptive analysis was performed with the gtsummary package, version 1.7.2 (2023‐07‐15) [20]. GraphPad Prism, version 9.5.1 for Windows, GraphPad Software, Boston, Massachusetts, USA, www.graphpad.com, and R were used for graphical visualization. Map diagrams were created using Microsoft Excel, version 2401. Whiskers in bar charts represent the 95%‐confidence interval.

For descriptive analysis, we used Pearson's Chi^2^‐Test or Fisher's Exact Test for categorical data and Wilcoxon Rank‐Sum Test for non‐normally distributed numeric parameters. Relative frequencies were calculated among cases with available information for the respective variable.

## 3 Results

### 3.1 Case Identification

**FIGURE 1:**
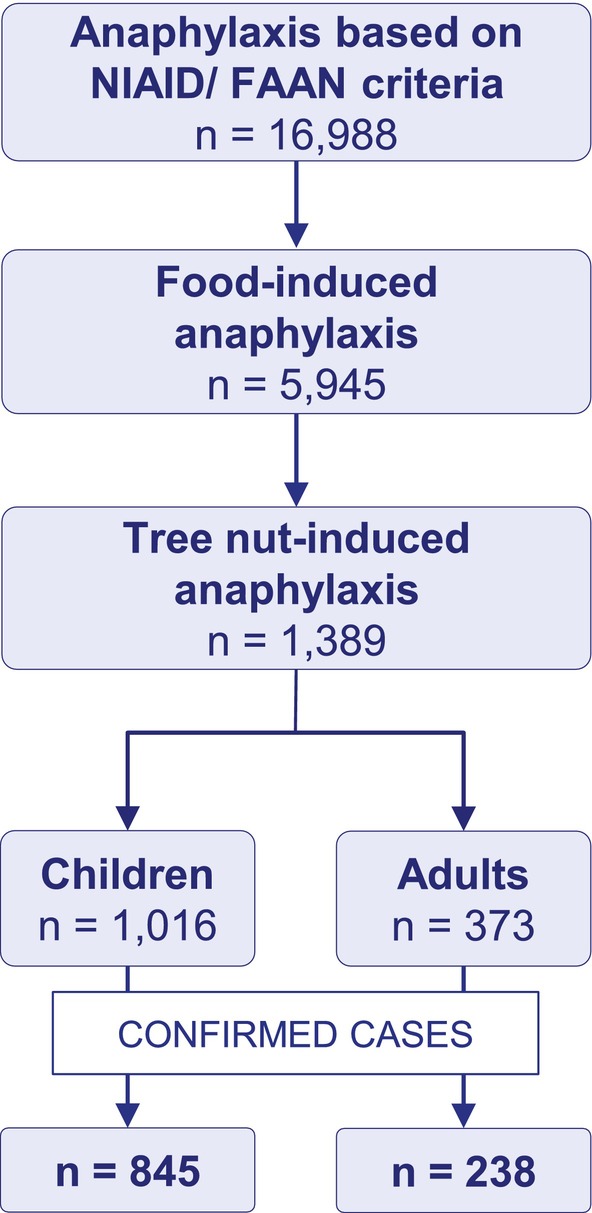
Flow chart depicting the selection process of tree nut‐induced anaphylaxis among cases from the European Anaphylaxis Registry. NIAID/FAAN: National Institute of Allergy and Infectious Diseases/Food Allergy and Anaphylaxis Network.

The EAR consists of 16,988 cases meeting the definition of anaphylaxis based on the NIAID/FAAN criteria (Figure 1). 5945 cases were elicited by food, and of these 1389 cases were TIA (23%). The elicitor was confirmed in 1083 cases (845 children and 238 adults) or highly suspected (171 children and 135 adults). We included cases with confirmed elicitor status for further analysis.

### 3.2 Demographics

Table 1 shows the baseline characteristics of the cohort, separated by age groups. The median age of children with confirmed TIA is 4 years (interquartile range: 2–8 years) and 38 (26–52) years in adults. Male patients were more frequently affected among children and adolescents, while more female patients were reported in the adult cohort.

**TABLE 1:** Baseline characteristics of patients with tree nut‐induced anaphylaxis.

Characteristic	Children^a^, ^c^ (*n* = 845)	Adults^a^, ^c^ (*n* = 238)	*p* ^b^
Age in years	4 (2, 8)	38 (26, 52)	**< 0.001**
Sex			**< 0.001**
Female	330 (39%)	142 (60%)	
Male	515 (61%)	96 (40%)	
Comorbidities			
Atopic Dermatitis	273 (35%)	18 (8.1%)	**< 0.001**
Asthma	202 (27%)	47 (21%)	0.095
Allergic rhinitis	186 (25%)	115 (51%)	**< 0.001**
Other food allergies	225 (36%)	26 (16%)	**< 0.001**
Cardiovascular disease	7 (1%)	34 (15%)	**< 0.001**
Mastocytosis	1 (0%)	5 (2%)	**0.003**
Presence of cofactors (overall)	142 (17%)	118 (50%)	**< 0.001**
Number of cofactors			**< 0.001**
1	133 (16%)	89 (37%)	
2	8 (1%)	22 (9%)	
3	1 (0%)	5 (2%)	
4	0	2 (1%)	
Severity of the reaction (Ring & Messmer)			**0.045**
Grade II	601 (71%)	165 (69%)	
Grade III	242 (29%)	69 (29%)	
Grade IV	2 (0%)	4 (2%)	
Severity of the reaction (Brown)			**< 0.001**
Grade I	52 (6%)	6 (3%)	
Grade II	632 (75%)	153 (65%)	
Grade III	160 (19%)	77 (33%)	
Affected organ systems			
Skin	798 (95%)	218 (92%)	0.079
Gastrointestinal tract	524 (62%)	107 (46%)	**< 0.001**
Respiratory tract	698 (83%)	203 (86%)	0.3
Cardiovascular system	214 (26%)	109 (48%)	**< 0.001**
Biphasic reaction	55 (7%)	8 (4%)	0.092
Previous reaction to same allergen	197 (25%)	72 (33%)	**0.016**
Allergy known before reaction	174 (23%)	42 (21%)	0.6
Amount of allergen leading to the reaction			**< 0.001**
< 1 teaspoon	135 (36%)	12 (12%)	
1 teaspoon – 1 tablespoon	203 (55%)	50 (51%)	
> 1 tablespoon	33 (8.9%)	36 (37%)	

*Note:* bold values: *p* < 0.05.

^a^ Median (IQR), *n* (%).

^b^ Wilcoxon rank sum test, Pearson's Chi‐squared test, Fisher's exact test.

^c^ Relative frequencies calculated among cases with available information for the respective variable.

### 3.3 Elicitors of Tree Nut‐Induced Anaphylaxis

**FIGURE 2:**
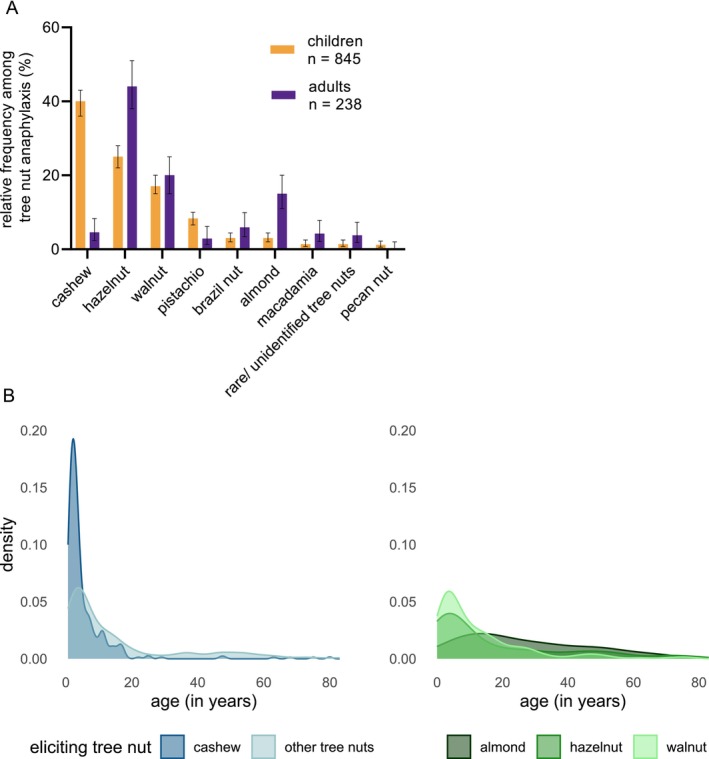
(A) Relative frequency of single tree nuts among cases of tree nut‐induced anaphylaxis, separated by age groups (children and adults). Whiskers indicate the 95%‐confidence intervals. (B) Age distribution of cashew‐, walnut‐, hazelnut‐, almond‐, and other tree nut‐induced anaphylaxis presented in density plots.

Among children with TIA, cashew (*n* = 334, 40%), hazelnut (*n* = 211, 25%) and walnut (*n* = 146, 17%) were the most frequent elicitors. Pistachio (*n* = 70, 8%), Brazil nut (*n* = 25, 3%), almond (*n* = 25, 3%), macadamia nut (*n* = 12, 1%), other rare/unidentified tree nuts (*n* = 12, 1%), and pecan nut (*n* = 10, 1%) were less frequent (Figure 2A).

In adults, hazelnut anaphylaxis accounted for 44% of all confirmed TIA (*n* = 105, *p*‐value for the comparison between children and adults: *p* < 0.001), followed by walnut (*n* = 47, 20%, *p* = 0.4) and almond (*n* = 35, 15%, *p* < 0.001). Less frequent elicitors of TIA in adults were Brazil nut (*n* = 14, 6%, *p* = 0.032), cashew (*n* = 11, 5%, *p* < 0.001), macadamia nut (*n* = 10, 4%, *p* = 0.015), other rare/unidentified tree nuts (*n* = 9, 4%, *p* = 0.030), and pistachio (*n* = 7, 3%, *p* = 0.005), while pecan nut was not reported among adults (*p* = 0.13) (Figure 2A). Data analyzed separately per tree nut can be found in the Tables S1–S9.

### 3.4 Age Distribution Among Cases of Anaphylaxis to Different Tree Nuts

The age‐dependent frequencies of TIA are shown in Figure 2B. Cashew‐induced anaphylaxis was most frequently reported among children younger than five years and rarely in patients older than ten years. Similar, but less pronounced observations were made among walnut‐induced anaphylaxis. By contrast, hazelnut‐induced anaphylaxis peaked in childhood and was registered throughout life even in adults > 80 years. Similarly, almond‐induced anaphylaxis and anaphylaxis to other tree nuts were observed throughout life with a slight predominance in childhood and young adulthood.

### 3.5 Comorbidities

The most common comorbidities in pediatric TIA were other food allergies (36%) and atopic dermatitis (35%), followed by asthma (27%) and allergic rhinitis (25%, Table 1). Among children with almond‐induced anaphylaxis, allergic rhinitis (59%) and asthma (41%) were more commonly reported than in children with anaphylaxis to other tree nuts (Table S5). Among adults, allergic rhinitis (51%), followed by asthma (21%) and additional food allergies (16%) were the most frequently reported comorbidities, while atopic dermatitis affected only 8% at the time of the reaction (Table 1). Non‐atopic comorbidities were rare in children but more frequent in adults, in particular cardiovascular diseases. Five adults and one child were diagnosed with mastocytosis.

### 3.6 Cofactors

Potential cofactors were suspected in 50% of adult TIA and 17% of pediatric reactions (*p* < 0.001). Also, more than one cofactor at the time of reaction was more often reported among adults (Table 1). In detail, exercise was the most frequent cofactor in children (14%), and drugs (31%) and exercise (21%) in adults. Among tree nuts, cofactors were observed in different frequencies: almond (36%), walnut (25%) and cashew (11%) in children (Supporting Information Tables 5 (almond), 3 (walnut) and 1 (cashew)). In adults, cofactors were reported in 70% of macadamia nut anaphylaxis and 63% of almond‐induced anaphylaxis (Supporting Information Tables 7 (macadamia) and 5 (almond)).

### 3.7 Reaction Severity and Biphasic Reactions

Most of the reported reactions to tree nuts were rated severity grade II based on Ring and Messmer's grading system [21]. Children and adults with TIA presented with comparable reaction severity (Table 1). Even though two grade IV reactions in children (induced by cashew and walnut) and four in adults were reported (all by hazelnut), none of these reactions had a fatal outcome. Teenagers presented with comparable reaction severity (data not shown).

According to Browns' severity grading system [22], 6% of children and 3% of adults had a grade I reaction (which results from the structure of the registry, that covers only reactions with respiratory and/or cardiovascular involvement), 75% of children and 65% of adults had a grade II reaction, and 19% of children and 33% of adults had a grade III reaction. Teenagers presented with a higher severity than children, but less severe reactions than adults (data not shown).

In the majority of TIA, skin symptoms occurred (> 90%), regardless of age. The second most frequently affected organ system was the respiratory system (both age groups), followed by gastrointestinal symptoms in children and cardiovascular symptoms in adults (Table 1).

Biphasic reactions were reported in 7% of children and 4% of adults with TIA (Table 1, *p* = 0.092, no statistical significance). They occurred in 81% within the first 12 h.

### 3.8 Cashew‐ and Pistachio‐Induced Anaphylaxis

As cashew and pistachio are highly cross‐reactive and patients often present with allergies towards both tree nuts [23], we compared patients with cashew and pistachio‐induced anaphylaxis (Table 2). Both groups showed highly similar features regarding their age and sex distribution, the presence of comorbidities, and cofactors. However, gastrointestinal symptoms occurred more frequently among cashew‐induced anaphylaxis. Reactions to cashew were significantly more severe than pistachio‐induced anaphylaxis when using Browns' severity grading system [22], but not according to Ring and Messmer [21].

**TABLE 2:** Comparison of patients with cashew‐ and pistachio‐induced anaphylaxis.

Characteristic	Cashew^a^, ^c^ (*n* = 345)	Pistachio^a^, ^c^ (*n* = 77)	*p* ^b^
Age in years	3.0 (2.0, 6.0)	4.0 (3.0, 9.0)	**0.005**
Age group children	334 (97%)	70 (91%)	**0.030**
Sex			> 0.9
Female	135 (39%)	30 (39%)	
Male	210 (61%)	47 (61%)	
Comorbidities			
Atopic dermatitis	108 (34%)	27 (39%)	0.5
Asthma	72 (23%)	20 (32%)	0.2
Allergic rhinitis	59 (20%)	19 (30%)	0.067
Other food allergies	86 (33%)	18 (33%)	> 0.9
Cardiovascular disease	7 (2%)	2 (3%)	0.7
Mastocytosis	1 (0%)	1 (2%)	0.3
Presence of cofactors (overall)	44 (13%)	15 (19%)	0.12
Number of cofactors			0.2
1	39 (11%)	13 (17%)	
2	3 (1%)	1 (1%)	
3	2 (1%)	0 (0%)	
4	0 (0%)	1 (1.3%)	
Severity of the reaction (Ring & Messmer)			0.8
Grade II	260 (75%)	60 (78%)	
Grade III	84 (24%)	17 (22%)	
Grade IV	1 (0%)	0 (0%)	
Severity of the reaction (Brown)			**0.009**
Grade I	15 (4%)	8 (10%)	
Grade II	252 (73%)	61 (79%)	
Grade III	78 (23%)	8 (10%)	
Affected organ systems			
Skin	322 (93%)	72 (95%)	0.8
Gastrointestinal tract	254 (74%)	46 (60%)	**0.015**
Respiratory tract	271 (79%)	65 (84%)	0.3
Cardiovascular system	98 (29%)	15 (20%)	0.11
Biphasic reaction	20 (6%)	8 (11%)	0.13
Previous reaction to same allergen	48 (15%)	9 (13%)	0.7
Allergy known before reaction	41 (13%)	13 (19%)	0.2
Amount of allergen leading to the reaction			0.6
< 1 teaspoon	52 (35%)	15 (42%)	
1 teaspoon – 1 tablespoon	84 (56%)	17 (47%)	
> 1 tablespoon	13 (9%)	4 (11%)	

*Note:* Bold values: *p* < 0.05.

^a^ Median (IQR), *n* (%).

^b^ Wilcoxon rank sum test, Pearson's Chi‐squared test, Fisher's exact test.

^c^ Relative frequencies calculated among cases with available information for the respective variable.

### 3.9 Amount of Allergen Eliciting the Anaphylactic Reaction and Time Until Reaction

**FIGURE 3:**
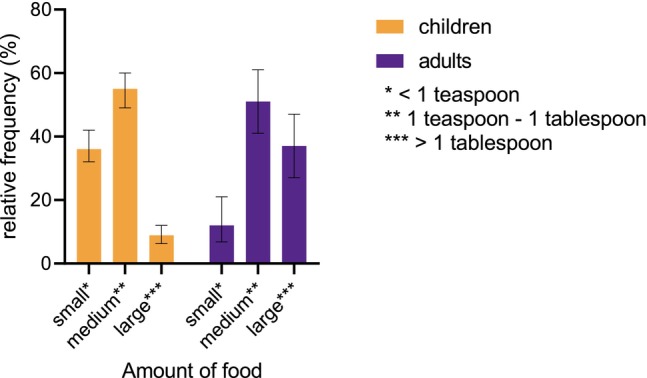
Amount of allergen‐containing food eliciting the reaction, separated by children and adults. Whiskers indicate the 95%‐confidence intervals.

TIA was mostly induced by small amounts of the allergen‐containing food. In the vast majority of cases, this amount was below or equal to one tablespoon (Figure 3). Overall, children reacted to smaller amounts than adults: in cashew‐induced anaphylaxis, 76% of children and 20% of adults reacted to amounts lower than one tablespoon (*p* = 0.015). Similar results were presented for hazelnut (59% in children, 27% in adults, *p* < 0.001), walnut (77% in children, 30% in adults, *p* < 0.001) and other tree nuts (76% in children, 28% in adults, *p* < 0.001).

Among children, 36% reacted to less than a teaspoon, and at least six pediatric reactions were elicited by inhalation or skin contact (cashew (*n* = 3), pistachio (*n* = 1) and walnut (*n* = 2)).

The time between exposure and onset of symptoms was age‐dependent. In 67% of children, the reaction occurred within the first ten minutes and in 96% within less than one hour after allergen contact. Among adults, only 42% reacted within the first ten minutes but 92% within the first hour (*p* < 0.001).

### 3.10 Previous Allergic Reactions and Awareness of the Allergy

A previous reaction to the allergen causing the anaphylactic reaction was reported among 25% of children and 33% of adults. Despite that, only 23% of children and 21% of adults were aware of their tree nut allergy beforehand.

### 3.11 Adrenaline Use, Hospitalization and Long‐Term Management

Among patients with a lay treatment, 47 children (13%) and 1 adult (3%) used an AAI. A voluntary question about the availability of an AAI before the reaction was introduced later and revealed that at least 111 children and eleven adults were prescribed an AAI before the reaction, which would suggest a frequency of 42% of children and 9% of adults using the available AAI during the reaction. However, these numbers have to be interpreted with great caution, because it might lead to an overestimation of the actual AAI use as eventually more patients were prescribed AAIs.

During professional first‐line treatment, 42% of children and 39% of adults received adrenaline. Adrenaline was used slightly more frequently if the reported reaction was a repetitive reaction instead of a first reaction: during lay treatment, adrenaline was used in 18% of repetitive and 9% of first reactions (*p* = 0.020). During professional treatment, adrenaline was used in 44% of repetitive and 41% of first reactions (*p* = 0.5 (no statistical significance), see Figure S1). 50% of children and 37% of adults were hospitalized due to the anaphylactic reaction. After the reaction, 780 children (97%) and 190 adults (88%) received a prescription for at least one AAI.

### 3.12 Reporting Countries

Anaphylactic reactions to tree nuts were mostly reported from Germany (*n* = 413), France (*n* = 270) and Switzerland (*n* = 144), which is in line with the general distribution of food‐induced anaphylaxis among the EAR. Walnut was the most frequent eliciting tree nut (among all TIA cases in the respective country) in Spain, and walnut and pistachio were the most frequent elicitors in Bulgaria. Hazelnut was most frequently reported from Germany, Austria, Switzerland, and Italy. Cases reported from Brazil were all elicited by Brazil nut. Cashew was the most frequently reported tree nut from France, Greece, Ireland, and Poland; see Figure S2.

### 3.13 Development of TIA Reports to the EAR over Time

**FIGURE 4:**
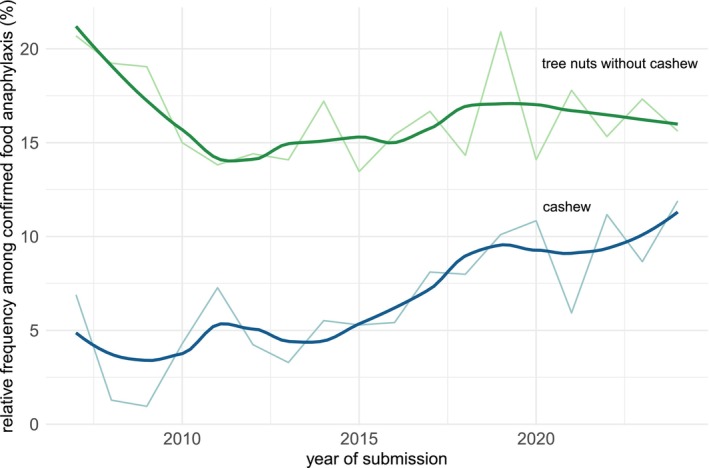
Relative frequency of cashew‐induced and tree nut excluding cashew‐induced anaphylaxis among all food‐induced anaphylaxis over time. Thin lines indicate exact annual frequencies; thick lines are smoothened.

To better understand the elicitor profile of TIA over time, we analyzed the relative frequency of cashew, hazelnut, walnut, and other tree nuts among all reported cases of food‐induced anaphylaxis from 2007 to 2024. As the absolute numbers of reactions are affected by the annual total number and activity of participating centres, we propose the relative frequency among food‐induced anaphylaxis as a proxy. There is a clear increase in the relative frequency of cashew‐induced anaphylaxis from 2007 to 2024 (year of report, Figure 4), while we do not observe clear trends for other tree nuts. The rising frequency of cashew‐induced anaphylaxis was still present if the analysis was restricted to reports from Germany, Austria, and Switzerland, as the registry was initially limited to German‐speaking centres and a country‐specific reporting profile might have influenced this observation (Figure S3).

## 4 Discussion

We report on > 1,000 cases of physician‐diagnosed, real‐life episodes of TIA based on data from the EAR. Tree nuts accounted for 23% of all reported food‐induced anaphylaxis, which confirms their role as one of the most important food allergens in Europe [24].

Cashew is the most frequent cause of TIA among children, while adults were rarely affected (334/345 cases, 97% pediatric cases). Similar observations in a smaller cohort were made among Swedish emergency department visits, where cashew elicited ten of 24 cases of TIA [25]. Patients were mostly younger than five years, previously unaware of their allergy and reacted quickly after the ingestion of small amounts. Cashew allergy is suggested to be associated with a higher risk of developing anaphylaxis compared to peanut allergy in children [26, 27] and very small amounts can cause allergic reactions [28, 29, 30]; its eliciting dose (ED)05 is only 0.8 mg protein compared to 2.1 mg peanut protein in affected patients [31]. Even reactions after skin/mucosal contact or inhalation have been reported [27, 32] and were also observed among three children in our cohort. Despite the low ED05 reported for some tree nuts (cashew: 0.8 mg, walnut: 0.8 mg, hazelnut: 3.5 mg) [31], TIA within the EAR was more frequently a first reaction to the respective allergen compared with peanuts or other food allergens (data from the EAR, not shown in this manuscript). This finding is consistent with other, yet smaller cohorts [32, 33] and might relate to the mandatory labeling of tree nuts on labels and menues [5].

We observed a clear increasing rate of cashew‐induced anaphylaxis among reported reactions to food. Cashew has been less widespread and relatively easy to avoid in the past [32]. Today, cashew is much more frequently consumed [34], e.g., as snacks, in cooked meals, but also as vegan milk‐ and meat‐substitute and in pesto [35]. The increased consumption likely affected increased sensitisation and reaction rates. The Finish Anaphylaxis Registry reported five cases from 2008 to 2012 and 29 cases from 2013 to 2017 [36]. A Swedish emergency department reported 20 cashew‐induced anaphylactic reactions between 2001 and 2010 [37]. These reports suggest an increase and support our findings from a large cohort. This trend should be monitored in the upcoming years and highlights the need for treatment options, as natural development of tolerance does not seem to occur frequently for this allergen (9%–14% of the cases) [14] and there is currently no approved treatment available. Oral immunotherapy (OIT) with tree nuts has been investigated in various clinical studies [38, 39, 40, 41], but has not been approved by the authorities so far. Patients with allergic reactions to cashew and pistachio showed very similar characteristics, which is in line with the known high cross‐reactivity of both nuts [23].

As described previously, hazelnuts and walnuts were frequent elicitors among children and adults in our cohort. Both of these tree nuts contain various allergenic proteins, including storage proteins, LTP, and PR‐10 proteins [6]. By contrast, storage proteins are identified as the predominant allergens in cashews [42], while a recently discovered PR‐10 protein [43] does not seem to be clinically relevant for patients with pollen‐associated food allergies [13]. Storage protein‐related tree nut allergies are responsible for the majority of pediatric cases of tree nut allergy, while adults from central Europe suffer mostly from PR‐10 protein‐ and adults from southern Europe from LTP‐related tree nut allergies. PR‐10 protein‐mediated allergies often cause milder symptoms like oral pruritus [13, 15], but can also induce severe reactions, in particular in the presence of cofactors and ingestion of larger amounts [12, 13, 44, 45]. Both larger amounts and a high frequency of potential cofactors were reported among the adults in our cohort. This points towards an intended ingestion of the nuts, which is in line with almost 80% of the patients being previously unaware of their allergy. Therefore, we suspect also PR‐10 protein‐ or LTP‐mediated tree nut anaphylaxis in our cohort, also for the patients with almond‐induced anaphylaxis, where we observed 25 pediatric and 35 adult cases, despite severe almond allergy being considered rare compared to other tree nut allergies [23, 46, 47]. The EAR does not provide information about the molecular sensitization profiles of the patients yet. But as almond contributed especially in Italy to the reports of TIA, it suggests a potential underlying LTP sensitization [48]. Atopic comorbidities were frequent (in line with other cohorts of TIA [49, 50, 51, 52]) and over 50% of the adult cohort suffered from concomitant allergic rhinitis, which might indicate PR10‐mediated anaphylaxis in some cases [12, 13, 44, 45]. Regional differences among the EAR might also be influenced by different dietary habits and the age distribution of reported cases within a country.

The majority of TIA in our cohort were of moderate severity according to Ring and Messmer and Brown's severity grading systems. Only six patients suffered from cardiac and/or respiratory arrest; none of them had a fatal outcome. This contrasts with previous reports, where tree nuts along with peanuts and cow's milk were frequent elicitors of fatal food‐induced anaphylaxis [53, 54]. As the EAR collects data from patients presenting in specialized centres after experiencing the reaction, such cases might be missed. However, overall, a fatal outcome of food‐induced anaphylaxis is a rare event [54, 55, 56] with a stable incidence over the last years [57].

We present data on more than 1000 physician‐reported real‐life cases of TIA. The data is derived from specialized allergy centers, which ensures high data quality, but might lead to a bias towards more severe or initial reactions to a specific allergen. An under‐representation of adults cannot be excluded, and the prevalence of TIA in general is not reflected. The structure of the EAR prevents us from using newer, food allergy‐specific severity‐grading systems such as the criteria presented from the World Allergy Organization [58]. However, tree nuts resemble the largest elicitor group among all food‐induced reactions in the EAR, and cashew‐induced anaphylaxis is on the rise among pediatric patients. Our data highlight that both pediatric and adult patients were in approximately 80% unaware of their allergy prior to the reaction, which is higher compared with other elicitor groups or food‐induced reactions in the EAR. Whether TIA might become even more frequent in the future, as tree nut allergy is often persistent and tree nut consumption increases due to changes in eating habits towards plant‐based protein sources, requires further monitoring. Considering the large number of patients, it is crucial to improve the management. Primary prevention to avoid tree nut allergy in general is necessary, followed by the education of patients about the potential role of cofactors, which were present in up to 50% of affected adults and which might have increased the reaction severity from otherwise mild to anaphylactic reactions. The prompt use of adrenaline during lay and professional treatment should be strongly advocated, and ultimately more research on treatment options such as immunotherapy or biologics for pediatric and adult patients is urgently required.

## Author Contributions

V.H. performed the statistical analysis and wrote the manuscript. S.D.‐B. coordinates the European Anaphylaxis Registry, performed the data cleaning and critically reviewed the manuscript. D.S.‐L. participated in data collection and critically reviewed the manuscript. A.D.‐C. participated in data collection and critically reviewed the manuscript. A.K. participated in data collection and critically reviewed the manuscript. M.B. participated in data collection and critically reviewed the manuscript. K.H. participated in data collection and critically reviewed the manuscript. L.L. participated in data collection and critically reviewed the manuscript. N.P. participated in data collection and critically reviewed the manuscript. H.O. participated in data collection and critically reviewed the manuscript. S.H. participated in data collection and critically reviewed the manuscript. M.B.B. participated in data collection and critically reviewed the manuscript. B.E.G. participated in data collection and critically reviewed the manuscript. M.W. established and maintains the European Anaphylaxis Registry, supervised the analysis, critically reviewed the initial draft and assisted in writing the manuscript.

## Conflicts of Interest

V.H. has no COI in relation to this paper. S.D.‐B. has no COI in relation to this paper. D.S.‐L. has no COI in relation to this paper. A.D.‐C. outside of the submitted work, reports grants from Don du Souffle, Novartis, ARAIRLOR, consulting fees from Sanofi, Stallergens, ALK, Aimmune Therapeutics, payment for presentations for Aimmune Therapeutics, Novartis, ALK, support for attending meetings from Mead Johnson, Nutricia, Aimmune Therapeutics, Novartis, ALK, stocks from Essilor Luxottica. A.K. has no COI in relation to this paper. M.B. has no COI in relation to this paper. K.H. received research funding from the Swiss National Science Foundation (SNSF; grant 310030_207705), the Swiss Cancer Research Foundation (grant KFS‐5979‐08‐2023) and the EU‐H2020‐MSCA‐COFUND EURIdoc programme (No. 101034170). K.H. is or recently was a speaker/advisor for ALK, Allergopharma, Almirall, BioCryst, Blueprint, Cogent, Galderma, KalVista, Leo, Menarini, Novartis, Pfizer, Sanofi, Takeda and ThermoFisher. L.L. has received speaker fees from DBV, Nutricia, Nestle, and Thermofisher Scientific. N.P. has received speaker/advisor fees from Abbott, Abbvie, ALK, Asit Biotech, AstraZeneca, Biomay, Boehringer Ingelheim, GSK, HAL, Faes Farma, Medscape, Menarini, MSD, Novartis, Nutricia, OM Pharma, Regeneron, Sanofi, Takeda, and Viatris. H.O. has no COI in relation to this paper. S.H. has no COI in relation to this paper. M.B.B. has no COI in relation to this paper. B.E.G. has no COI in relation to this paper. M.W. declares the receipt of honoraria or consultation fees by the following companies: Abbvie, Aimmune, ALK‐Abelló, Allergopharma, Almirall, Amgen, AstraZeneca, Bayer, Bencard, Bioprojet Pharma, Bristol‐Myers Squibb, Boehringer Ingelheim, Galderma, Glaxosmithkline, Infectopharm, Leo Pharma, Eli Lilly, Mylan/Viatris, Novartis, Octapharma, Pfizer and Sanofi.

## Supporting information


Figure S1.

Table S1.

## Data Availability

The data that support the findings of this study are available from the corresponding author upon reasonable request.
