# Supplementary material for: A Growing Concern for Cashew and an Unexpected Risk From Almonds: Data From the Anaphylaxis Registry
Source: Allergy. 2025 Jun 13;80(10):2837–48. doi: 10.1111/all.16619 (PMC12486339; doi:10.1111/all.16619)
Supplement: Supplementary file 1 — Figure S1. [file ALL-80-2837-s001.pdf]

**Supplemental Figures**

Supplemental Figure 1: Use of adrenaline during anaphylactic reactions to tree nuts in first and repetitive reactions among patients who received a lay or professional treatment, respectively. Among 594 children with a first reaction to this elicitor, 141 were treated only by a lay person, 325 only by a professional person, 79 by lay followed by a professional. 35 children received no treatment, for 14 cases information was missing. Among 146 adults with a first reaction, 14 received lay treatment only, 111 professional treatment only, five both, 13 no treatment, three missing answers.

Among 197 children with repetitive reactions to this elicitor, 86 were treated by a lay person only, 70 by professionals only, 28 by both, eleven no treatment, two missing information. Among 72 adults with repetitive reactions, ten received only a lay treatment, 50 only professional, three both, five no treatment, four missing answers. For 54 children and 20 adults, information about previous allergic reactions were unavailable. Those were excluded from this analysis.

Whiskers indicate the 95%-confidence intervals.

Supplemental Figure 2: Relative frequency of tree nut-specific anaphylaxis among all TIA cases in the respective reporting European countries (calculated as the frequency of anaphylaxis to the specific tree nut divided by all TIA cases with confirmed elicitor status of the respective country). Darker colours indicate a higher relative frequency.

Supplemental Figure 3: Relative frequency of cashew-induced and tree nut excluding cashew-induced anaphylaxis among all food-induced anaphylaxis over time, limited to reports from Germany, Austria and Switzerland. Thin lines indicate exact annual frequencies, thick lines are smoothened.

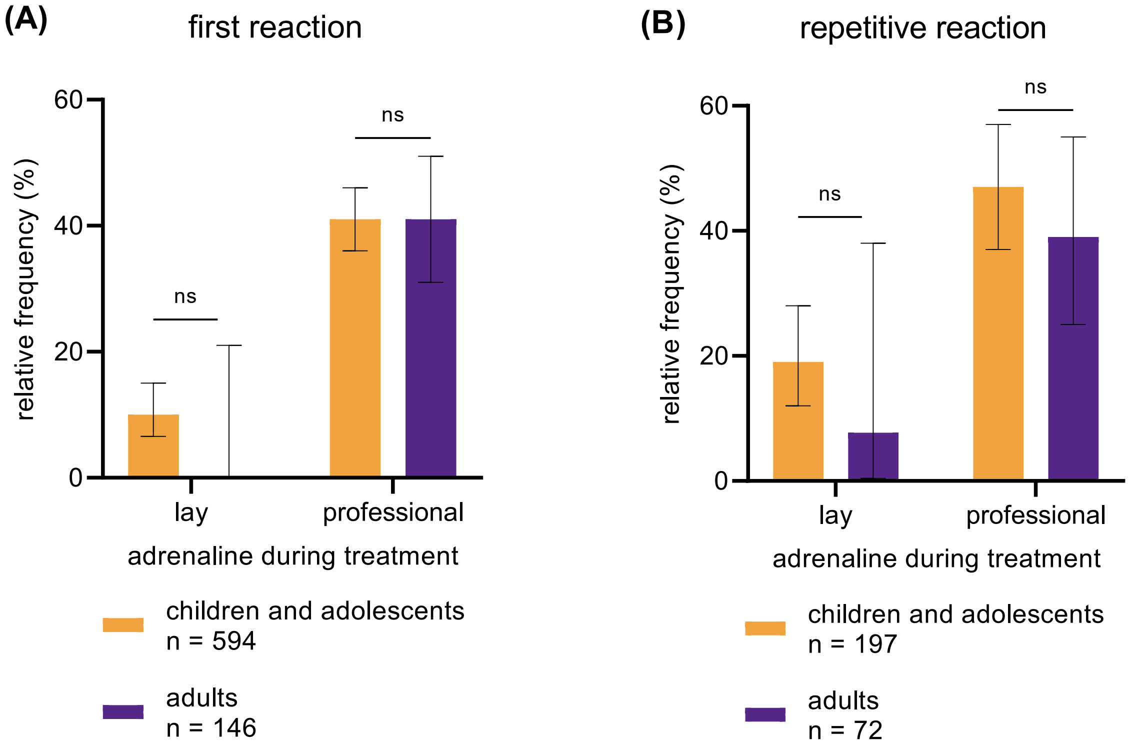


Supplemental Figure 1_Höfer et al.

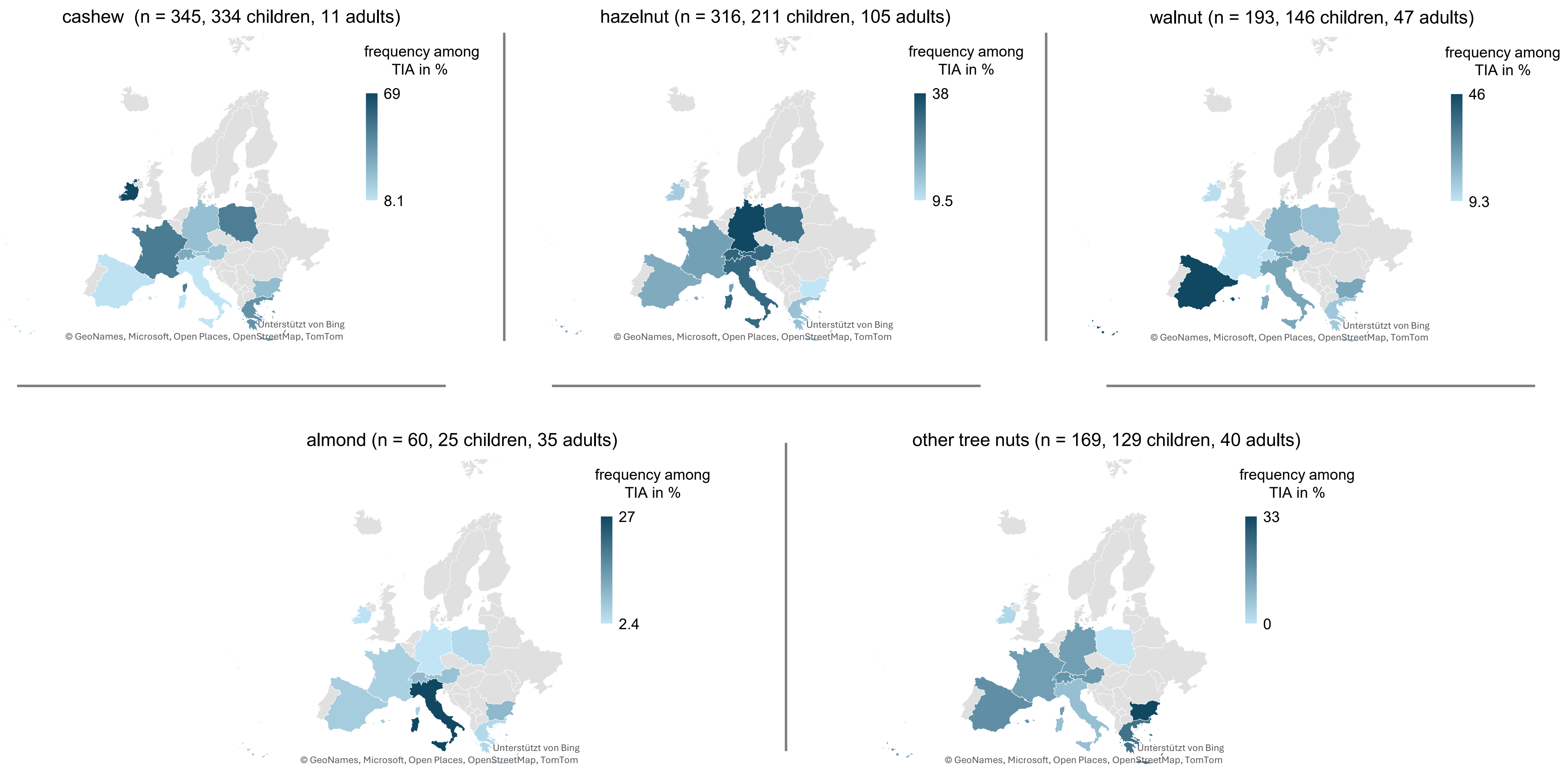


Supplemental Figure 2_Höfer et al.

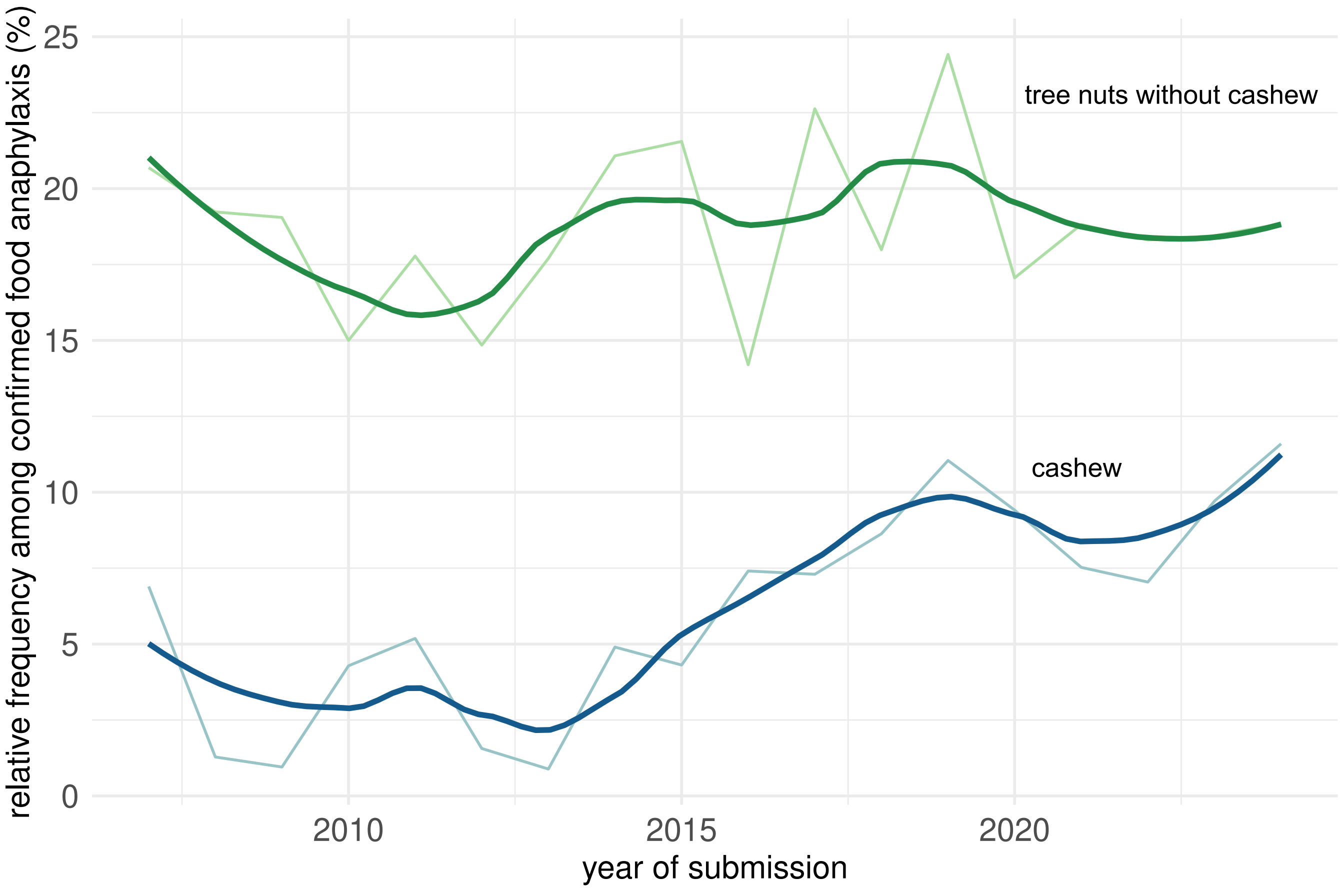


Supplemental Figure 3_Höfer et al.
