# Supplementary material for: A Growing Concern for Cashew and an Unexpected Risk From Almonds: Data From the Anaphylaxis Registry
Source: Allergy. 2025 Jun 13;80(10):2837–48. doi: 10.1111/all.16619 (PMC12486339; doi:10.1111/all.16619)
Supplement: Supplementary file 2 — Table S1. [file ALL-80-2837-s002.pdf]

Supplement Table 1: Baseline characteristics of patients with cashew-induced anaphylaxis
†Median (IQR); n (%), §Relative frequencies calculated among cases with available information for the respective variable

**Characteristic**	**children/adolescents**, N = 334†, §	**adults**, N = 11†, §
age in years	3.0 (2.0, 6.0)	47.0 (25.0, 65.5)
sex		
female	127 (38%)	8 (73%)
male	207 (62%)	3 (27%)
comorbidities		
atopic dermatitis	107 (35%)	1 (9%)
asthma	68 (23%)	4 (36%)
allergic rhinitis	54 (19%)	5 (45%)
other food allergy	84 (33%)	2 (33%)
cardiovascular disease	4 (1%)	3 (27%)
mastocytosis	1 (0%)	0 (0%)
presence of cofactors (overall)	38 (11%)	6 (55%)
number of cofactors		
1	34 (10%)	5 (45%)
2	3 (1%)	0 (0%)
3	1 (0%)	1 (9%)
severity of the reaction (Ring and Messmer)		
grade II	254 (76%)	6 (55%)
grade III	79 (24%)	5 (45%)
grade IV	1 (0%)	0 (0%)
severity of the reaction (Brown)		
grade I	15 (5%)	0 (0%)
grade II	247 (74%)	5 (45%)
grade III	72 (22%)	6 (55%)
affected organ system		
skin	313 (94%)	9 (82%)
gastrointestinal tract	246 (74%)	8 (73%)
respiratory tract	263 (79%)	8 (73%)
cardiovascular system	93 (28%)	5 (45%)
biphasic reaction	20 (6%)	0 (0%)
previous reaction to the same allergen	45 (14%)	3 (33%)
allergy known before reaction	39 (13%)	2 (18%)
amount of allergen leading to the reaction		
<1 teaspoon	52 (36%)	0 (0%)
1 teaspoon - 1 tablespoon	82 (57%)	2 (40%)
>1 tablespoon	10 (7%)	3 (60%)

Supplement Table 2: Baseline characteristics of patients with hazelnut-induced anaphylaxis
†Median (IQR); n (%), §Relative frequencies calculated among cases with available information for the respective variable

**Characteristic**	**children/adolescents**, N = 211†, §	**adults**, N = 105†, §
age in years	3 (2, 8)	43 (29, 54)
sex		
female	80 (38%)	64 (61%)
male	131 (62%)	41 (39%)
comorbidities		
atopic dermatitis	77 (39%)	9 (9.2%)
asthma	54 (28%)	22 (22%)
allergic rhinitis	48 (25%)	56 (57%)
other food allergy	56 (37%)	14 (19%)
cardiovascular disease	3 (2%)	17 (17%)
mastocytosis	0 (0%)	4 (4%)
presence of cofactors (overall)	38 (18%)	54 (52%)
number of cofactors		
1	34 (16%)	40 (38%)
2	4 (2%)	11 (10%)
3	0 (0%)	2 (2%)
4	0 (0%)	1 (1.0%)
severity of the reaction (Ring and Messmer)		
grade II	145 (69%)	71 (68%)
grade III	66 (31%)	30 (29%)
grade IV	0 (0%)	4 (4%)
severity of the reaction (Brown)		
grade I	15 (7%)	3 (3%)
grade II	161 (76%)	67 (65%)
grade III	35 (17%)	33 (32%)
affected organ system		
skin	200 (95%)	96 (91%)
gastrointestinal tract	113 (54%)	44 (42%)
respiratory tract	180 (86%)	93 (89%)
cardiovascular system	51 (25%)	49 (49%)
biphasic reaction	11 (6%)	5 (5%)
previous reaction to the same allergen	91 (46%)	34 (34%)
allergy known before reaction	79 (43%)	19 (22%)
amount of allergen leading to the reaction		
<1 teaspoon	29 (29%)	5 (12%)
1 teaspoon - 1 tablespoon	57 (58%)	21 (51%)
>1 tablespoon	13 (13%)	15 (37%)

Supplement Table 3: Baseline characteristics of patients with walnut-induced anaphylaxis
†Median (IQR); n (%), §Relative frequencies calculated among cases with available information for the respective variable

Characteristic	children/adolescents, N = 146†, §	adults, N = 47†, §
age in years	5 (3, 9)	27 (22, 44)
sex		
female	64 (44%)	28 (60%)
male	82 (56%)	19 (40%)
comorbidities		
atopic dermatitis	41 (30%)	5 (12%)
asthma	32 (24%)	10 (23%)
allergic rhinitis	32 (24%)	25 (57%)
other food allergy	42 (37%)	1 (3%)
cardiovascular disease	0 (0%)	2 (5%)
mastocytosis	0 (0%)	0 (0%)
presence of cofactors (overall)	36 (25%)	17 (36%)
number of cofactors		
1	36 (25%)	12 (26%)
2	0 (0%)	4 (9%)
3	0 (0%)	1 (2%)
severity of the reaction (Ring and Messmer)		
grade II	88 (60%)	31 (66%)
grade III	57 (39%)	16 (34%)
grade IV	1 (1%)	0 (0%)
severity of the reaction (Brown)		
grade I	11 (8%)	2 (4%)
grade II	104 (71%)	30 (64%)
grade III	30 (21%)	15 (32%)
affected organ system		
skin	142 (97%)	43 (91%)
gastrointestinal tract	74 (52%)	25 (53%)
respiratory tract	129 (89%)	36 (78%)
cardiovascular system	35 (24%)	25 (58%)
biphasic reaction	11 (8%)	1 (3%)
previous reaction to the same allergen	31 (23%)	15 (38%)
allergy known before reaction	31 (23%)	13 (31%)
amount of allergen leading to the reaction		
<1 teaspoon	31 (48%)	4 (20%)
1 teaspoon - 1 tablespoon	29 (45%)	8 (40%)
>1 tablespoon	4 (6%)	8 (40%)

Supplement Table 4: Baseline characteristics of patients with pistachio-induced anaphylaxis
†Median (IQR); n (%), §Relative frequencies calculated among cases with available information for the respective variable

**Characteristic**	**children/adolescents**, N = 70†, §	**adults**, N = 7†, §
age in years	4 (3, 7)	37 (33, 42)
sex		
female	26 (37%)	4 (57%)
male	44 (63%)	3 (43%)
comorbidities		
atopic dermatitis	26 (41%)	1 (17%)
asthma	19 (33%)	1 (17%)
allergic rhinitis	18 (32%)	1 (17%)
other food allergy	18 (36%)	0 (0%)
cardiovascular disease	0 (0%)	2 (29%)
mastocytosis	0 (0%)	1 (14%)
presence of cofactors (overall)	11 (16%)	4 (57%)
number of cofactors		
1	11 (16%)	2 (29%)
2	0 (0%)	1 (14%)
4	0 (0%)	1 (14%)
severity of the reaction (Ring and Messmer)		
grade II	56 (80%)	4 (57%)
grade III	14 (20%)	3 (43%)
grade IV	0 (0%)	0 (0%)
severity of the reaction (Brown)		
grade I	8 (11%)	0 (0%)
grade II	55 (79%)	6 (86%)
grade III	7 (10%)	1 (14%)
affected organ system		
skin	65 (94%)	7 (100%)
gastrointestinal tract	43 (61%)	3 (43%)
respiratory tract	58 (83%)	7 (100%)
cardiovascular system	13 (19%)	2 (29%)
biphasic reaction	8 (12%)	0 (0%)
previous reaction to the same allergen	7 (11%)	2 (29%)
allergy known before reaction	12 (19%)	1 (17%)
amount of allergen leading to the reaction		
<1 teaspoon	15 (47%)	0 (0%)
1 teaspoon - 1 tablespoon	14 (44%)	3 (75%)
>1 tablespoon	3 (9%)	1 (25%)

Supplement Table 5: Baseline characteristics of patients with almond-induced anaphylaxis
†Median (IQR); n (%), §Relative frequencies calculated among cases with available information for the respective variable

**Characteristic**	**children/adolescents**, N = 25†, §	**adults**, N = 35†, §
age in years	10 (6, 14)	34 (27, 50)
sex		
female	9 (36%)	22 (63%)
male	16 (64%)	13 (37%)
comorbidities		
atopic dermatitis	4 (20%)	1 (3%)
asthma	9 (41%)	5 (14%)
allergic rhinitis	13 (59%)	13 (37%)
other food allergy	11 (58%)	4 (17%)
cardiovascular disease	0 (0%)	6 (19%)
mastocytosis	0 (0%)	0 (0%)
presence of cofactors (overall)	9 (36%)	22 (63%)
number of cofactors		
1	9 (36%)	16 (46%)
2	0 (0%)	5 (14%)
3	0 (0%)	1 (3%)
severity of the reaction (Ring and Messmer)		
grade II	18 (72%)	30 (86%)
grade III	7 (28%)	5 (14%)
grade IV	0 (0%)	0 (0%)
severity of the reaction (Brown)		
grade I	0 (0%)	1 (3%)
grade II	20 (80%)	24 (69%)
grade III	5 (20%)	10 (29%)
affected organ system		
skin	22 (92%)	31 (89%)
gastrointestinal tract	14 (56%)	14 (44%)
respiratory tract	19 (76%)	30 (86%)
cardiovascular system	8 (32%)	12 (36%)
biphasic reaction	1 (5%)	1 (3%)
previous reaction to the same allergen	10 (45%)	13 (39%)
allergy known before reaction	8 (36%)	6 (20%)
amount of allergen leading to the reaction		
<1 teaspoon	1 (11%)	2 (12%)
1 teaspoon - 1 tablespoon	7 (78%)	8 (47%)
>1 tablespoon	1 (11%)	7 (41%)

Supplement Table 6: Baseline characteristics of patients with Brazil nut-induced anaphylaxis
†Median (IQR); n (%), §Relative frequencies calculated among cases with available information for the respective variable

**Characteristic**	**children/adolescents**, N = 25†, §	**adults**, N = 14†, §
age in years	5 (3, 12)	42 (25, 56)
sex		
female	11 (44%)	5 (36%)
male	14 (56%)	9 (64%)
comorbidities		
atopic dermatitis	6 (26%)	0 (0%)
asthma	4 (18%)	2 (17%)
allergic rhinitis	5 (22%)	5 (42%)
other food allergy	2 (11%)	3 (38%)
cardiovascular disease	0 (0%)	2 (17%)
mastocytosis	0 (0%)	0 (0%)
presence of cofactors (overall)	6 (24%)	3 (21%)
number of cofactors		
1	5 (20%)	3 (21%)
2	1 (4%)	0 (0%)
severity of the reaction (Ring and Messmer)		
grade II	20 (80%)	10 (71%)
grade III	5 (20%)	4 (29%)
grade IV	0 (0%)	0 (0%)
severity of the reaction (Brown)		
grade I	1 (4%)	0 (0%)
grade II	17 (68%)	9 (64%)
grade III	7 (28%)	5 (36%)
affected organ system		
skin	25 (100%)	13 (93%)
gastrointestinal tract	13 (52%)	6 (46%)
respiratory tract	20 (83%)	14 (100%)
cardiovascular system	8 (33%)	6 (46%)
biphasic reaction	2 (9%)	0 (0%)
previous reaction to the same allergen	4 (17%)	2 (15%)
allergy known before reaction	1 (4%)	0 (0%)
amount of allergen leading to the reaction		
<1 teaspoon	5 (38%)	1 (14%)
1 teaspoon - 1 tablespoon	7 (54%)	5 (71%)
>1 tablespoon	1 (8%)	1 (14%)

Supplement Table 7: Baseline characteristics of patients with macadamia nut-induced anaphylaxis
†Median (IQR); n (%), §Relative frequencies calculated among cases with available information for the respective variable

**Characteristic**	**children/adolescents**, N = 12†, §	**adults**, N = 10†, §
age in years	7 (4, 10)	55 (50, 62)
sex		
female	5 (42%)	6 (60%)
male	7 (58%)	4 (40%)
comorbidities		
atopic dermatitis	2 (18%)	1 (11%)
asthma	5 (45%)	2 (22%)
allergic rhinitis	6 (50%)	5 (56%)
other food allergy	6 (60%)	1 (25%)
cardiovascular disease	0 (0%)	2 (22%)
mastocytosis	0 (0%)	0 (0%)
presence of cofactors (overall)	2 (17%)	7 (70%)
number of cofactors		
1	2 (17%)	7 (70%)
severity of the reaction (Ring and Messmer)		
grade II	8 (67%)	7 (70%)
grade III	4 (33%)	3 (30%)
grade IV	0 (0%)	0 (0%)
severity of the reaction (Brown)		
grade I	2 (17%)	0 (0%)
grade II	9 (75%)	9 (90%)
grade III	1 (8%)	1 (10%)
affected organ system		
skin	11 (92%)	10 (100%)
gastrointestinal tract	7 (58%)	1 (10%)
respiratory tract	11 (92%)	9 (90%)
cardiovascular system	2 (17%)	3 (30%)
biphasic reaction	1 (9%)	0 (0%)
previous reaction to the same allergen	2 (17%)	1 (13%)
allergy known before reaction	1 (9%)	0 (0%)
amount of allergen leading to the reaction		
<1 teaspoon	2 (33%)	0 (0%)
1 teaspoon - 1 tablespoon	4 (67%)	2 (67%)
>1 tablespoon	0 (0%)	1 (33%)

Supplement Table 8: Baseline characteristics of patients with anaphylaxis induced by rare/unidentified tree nuts

†Median (IQR); n (%), §Relative frequencies calculated among cases with available information for the respective variable

**Characteristic**	**children/adolescents**, N = 12†, §	**adults**, N = 9†, §
age in years	12 (6, 14)	34 (26, 45)
sex		
female	4 (33%)	5 (56%)
male	8 (67%)	4 (44%)
comorbidities		
atopic dermatitis	7 (58%)	0 (0%)
asthma	7 (58%)	1 (11%)
allergic rhinitis	8 (67%)	5 (56%)
other food allergy	1 (33%)	1 (20%)
cardiovascular disease	0 (0%)	0 (0%)
mastocytosis	0 (0%)	0 (0%)
presence of cofactors (overall)	1 (8%)	5 (56%)
number of cofactors		
1	1 (8%)	4 (44%)
2	0 (0%)	1 (11%)
severity of the reaction (Ring and Messmer)		
grade II	7 (58%)	6 (67%)
grade III	5 (42%)	3 (33%)
grade IV	0 (0%)	0 (0%)
severity of the reaction (Brown)		
grade II	8 (67%)	3 (33%)
grade III	4 (33%)	6 (67%)
affected organ system		
skin	10 (83%)	9 (100%)
gastrointestinal tract	7 (58%)	6 (67%)
respiratory tract	10 (83%)	6 (67%)
cardiovascular system	3 (25%)	7 (78%)
biphasic reaction	0 (0%)	1 (13%)
previous reaction to the same allergen	7 (64%)	2 (25%)
allergy known before reaction	2 (50%)	1 (17%)
amount of allergen leading to the reaction		
<1 teaspoon	0 (0%)	0 (0%)
1 teaspoon - 1 tablespoon	2 (100%)	1 (100%)
>1 tablespoon	0 (0%)	0 (0%)

Supplement Table 9: Baseline characteristics of patients with pecan nut-induced anaphylaxis
†Median (IQR); n (%), §Relative frequencies calculated among cases with available information for the respective variable

**Characteristic**	**children/adolescents**, N = 10†, §	**adults**, N = 0
age in years	5 (3, 6)	
sex		
female	4 (40%)	-
male	6 (60%)	-
comorbidities		
atopic dermatitis	3 (33%)	-
asthma	4 (40%)	-
allergic rhinitis	2 (22%)	-
other food allergy	5 (56%)	-
cardiovascular disease	0 (0%)	-
mastocytosis	0 (0%)	-
presence of cofactors (overall)	1 (10%)	-
number of cofactors		-
1	1 (10%)	
severity of the reaction (Ring and Messmer)		
grade II	5 (50%)	-
grade III	5 (50%)	-
grade IV	0 (0%)	-
severity of the reaction (Brown)		
grade II	10 (100%)	-
affected organ system		
skin	10 (100%)	-
gastrointestinal tract	7 (70%)	-
respiratory tract	8 (80%)	-
cardiovascular system	1 (10%)	-
biphasic reaction	1 (11%)	-
previous reaction to the same allergen		
no	9 (100%)	-
allergy known before reaction	1 (10%)	-
amount of allergen leading to the reaction		
<1 teaspoon	0 (0%)	-
1 teaspoon - 1 tablespoon	1 (50%)	-
>1 tablespoon	1 (50%)	-
